# Dual-source computed tomography of the lung with spectral shaping and advanced iterative reconstruction: potential for maximum radiation dose reduction

**DOI:** 10.1007/s00247-020-04714-0

**Published:** 2020-06-17

**Authors:** Matthias Wetzl, Matthias S. May, Daniel Weinmann, Matthias Hammon, Christoph Treutlein, Martin Zeilinger, Alexander Kiefer, Regina Trollmann, Joachim Woelfle, Michael Uder, Oliver Rompel

**Affiliations:** 1 Department of Radiology, University Hospital Erlangen, Erlangen, Germany; 2 Imaging Science Institute, University Hospital Erlangen, Erlangen, Germany; 3 Department of Pediatrics and Adolescent Medicine, University Hospital Erlangen, Erlangen, Germany

**Keywords:** Adolescents, Advanced iterative reconstruction, Children, Dual-source computed tomography, Tin prefiltration, Lung, Radiation dose reduction, Spectral shaping

## Abstract

**Background:**

Radiation dose at CT should be as low as possible without compromising diagnostic quality.

**Objective:**

To assess the potential for maximum dose reduction of pediatric lung dual-source CT with spectral shaping and advanced iterative reconstruction (ADMIRE).

**Materials and methods:**

We retrospectively analyzed dual-source CT acquisitions in a full-dose group (FD: 100 kV, 64 reference mAs) and in three groups with spectral shaping and differing reference mAs values (Sn: 100 kV, 96/64/32 reference mAs), each group consisting of 16 patients (age mean 11.5 years, standard deviation 4.8 years, median 12.8 years, range 1.3–18 years). Advanced iterative reconstruction of images was performed with different strengths (FD: ADMIRE Level 2; Sn: ADMIRE Levels 2, 3 and 4). We analyzed dose parameters and measured noise. Diagnostic confidence and detectability of lung lesions as well as anatomical structures were assessed using a Likert scale (from 1 [unacceptable] to 4 [fully acceptable]).

**Results:**

Compared to full dose, effective dose was reduced to 16.7% in the Sn 96 group, 11.1% in Sn64, and 5.5% in Sn32 (*P*<0.001). Noise values of Sn64_ADM4_ did not statistically differ from those in FD_ADM2_ (45.7 vs. 38.9 Hounsfield units [HU]; *P*=0.132), whereas noise was significantly higher in Sn32_ADM4_ compared to Sn64_ADM4_ (61.5 HU; *P*<0.001). A Likert score >3 was reached in Sn64_ADM4_ regarding diagnostic confidence (3.2) and detectability of lung lesions (3.3). For detectability of most anatomical structures, no significant differences were found between FD_AM2_ and Sn64_ADM4_ (*P*≥0.05).

**Conclusion:**

In pediatric lung dual-source CT, spectral shaping together with ADMIRE 4 enable radiation dose reduction to about 10% of a full-dose protocol while maintaining an acceptable diagnostic quality.

## Introduction

Dual-source CT is widely used for evaluating lung diseases in children and adolescents. In general, different approaches to decreasing CT radiation exposure have been proposed. One of the most effective methods is the reduction of tube voltage, because the dose increases with the square of the tube voltage. On the other hand, it varies approximately linearly with tube current [1].

Recently, third-generation dual-source CT scanners have been equipped with additional tin prefiltration that removes low-energy photons of the X-ray beam. These photons contribute little to image quality but increase radiation burden. The so-called spectral shaping has enabled radiation dose reduction in several anatomical regions in adults and children [2–5].

It has been reported that advanced iterative reconstruction enables reduction of radiation dose while preserving image quality in pediatric CT examinations [6]. Newell et al. [7] reported a phantom study indicating that third-generation dual-source CT scanners using third-generation iterative reconstruction methods (ADMIRE; Siemens Healthcare, Erlangen, Germany) can generate accurate quantitative CT images with acceptable image noise at very low dose levels. In a study of Rompel et al. [8], chest CT angiography in newborns and young children performed with a third-generation dual-source CT scanner using a 70-kV protocol together with stronger reconstruction levels of ADMIRE allowed high image quality at low radiation dose level.

We hypothesized that pediatric lung dual-source CT spectral shaping together with a strong reconstruction increment of ADMIRE would enable substantial radiation dose reduction while maintaining an acceptable diagnostic quality. Accordingly, the aim of this study was to identify the percentage value of possible dose reduction compared to a full-dose examination protocol.

## Materials and methods

We conducted this study in accordance with the guidelines of the Declaration of Helsinki; our local ethics committee approved the study. Written informed consent for dual-source CT of the lung was obtained for all patients. The institutional review board waived supplemental agreement because of the retrospective study design.

### Patient characteristics

A total of 64 patients with dual-source CT examinations of the lung were enrolled in this study. They were retrospectively selected from four examination protocols available in our department. In 16 patients (age 11.2±5.0 years, median 12.4 years, range 2.9–17.7 years) a full-dose (FD) dual-source CT of the lung had been conducted. Forty-eight other patients had been examined using one of three reduced-dose protocols with tin prefiltration (Sn) established in our department (Sn96: *n*=16, 10.3±6.1 years, median 11.6 years, range 1.3–17.7 years; Sn64: *n*=16, 13.1±3.4 years, median 13.1 years, range 5.6–18.0 years; Sn32: *n*=16, 11.4±4.2 years, median 12.8 years, range 4.8–17.6 years). The Sn protocols had been implemented at our institute in order to gradually reduce radiation exposure in clinical routine. Patients of the different groups were matched for age, weight and body mass index. Among all groups there was no significant difference in patient characteristics (Table 1).

**Table 1:** Patient characteristics of the different dose groups

Dose group	Full-dose	Sn96	Sn64	Sn32	*P*-value^a^
Number of patients	16	16	16	16	
Gender	9 male, 7 female	9 male, 7 female	11 male, 5 female	12 male, 4 female	
Age: mean±SD, median (range), years	11.2±5.0 12.4 (2.9–17.7)	10.3±6.1 11.6 (1.3–17.7)	13.1±3.4 13.1 (5.6–18.0)	11.4±4.2 12.8 (4.8–17.6)	ANOVA, *P*=0.435
Weight: mean±SD, kg	40.9±22.9	36.1±19.3	46.9±16.1	45.6±24.1	ANOVA, *P*=0.457
Body mass index: mean±SD	17.8±3.8	18.5±4.2	18.7±3.2	19.3±5.3	ANOVA, *P*=0.791

*ANOVA* analysis of variance, *SD* standard deviation, *Sn96/Sn64/Sn32* dose groups with tin prefiltration and different reference tube current–time products (96/64/32 reference mAs, respectively)

^a^*P*-value <0.05 is significant

All patients had been referred for CT to further investigate suspected or known non-cancer lung diseases such as cystic fibrosis, primary ciliary dyskinesia, prolonged course of pneumonia, chronic lung complications of pneumonia, suspected pulmonary hemorrhage, aspiration pneumonitis, pulmonary Langerhans cell histiocytosis, tuberculosis, and atelectasis or pleural effusion of unclear origin.

### Dual-source computed tomography techniques

All dual-source CT examinations were performed using the same third-generation scanner (Somatom Definition Force, Siemens Healthcare). CT parameters were as follows: 0.25 s gantry rotation time, detector collimation of 2x96x0.6 mm, slice collimation of 192×0.6 mm using z-flying focal spot technique, spiral pitch factor 3.0, tube voltage modulation switched off. In the full-dose protocol, patients were examined at a 100-kV setting with automatic exposure control (reference tube current time product per rotation 64 mAs; CareDose4D, Siemens Healthcare). In all other protocols 0.6-mm tin prefiltration was applied. Because tin prefiltration is only available at 100-kV and 150-kV tube voltage, with higher diagnostic dose efficiency at 100 kV [9], the lower kV setting is used in our department. For the three examination protocols with spectral shaping, default values of reference tube current–time product per rotation were 96 mAs/64 mAs/32 mAs. Examinations were performed in supine position with elevated arms from the upper to the lower thoracic aperture. If necessary, a body-weight-adapted dose of iodinated contrast medium was injected intravenously (iomeprol 300 mg/mL, Iomeron, Bracco Imaging, Konstanz, Germany; or Accutron CT-D, Medtron AG, Saarbrücken, Germany).

### Postprocessing

Primary image data were automatically generated with a slice thickness of 0.6 mm using filtered back-projection (FBP). Additionally, all data sets of examination protocols including tin prefiltration were generated with advanced iterative reconstruction utilizing a medium, an intermediate and a strong increment (ADMIRE strengths 2/3/4). Slice thickness was 0.6 mm, in these protocols, too. In our clinical practice we observed adequate diagnostic quality on full-dose examinations when ADMIRE 2 was used. Consequently, reconstruction of ADMIRE 3 and ADMIRE 4 had not been performed at the time of examinations and thus was not available in the retrospective setting of this study. Iterative reconstruction is characterized by repeated forward and back projection of raw data and image data in combination with statistical modeling. The repeated comparison of projected raw data with the measured data allows removal of geometric imperfections. ADMIRE is built upon these principles, with substantial modifications, allowing a high iteration speed [7]. It has been shown that ADMIRE has the potential to significantly improve image quality while reducing noise and artifacts in CT scans [8, 10, 11]. In ADMIRE, images are reconstructed by minimizing the objective function incorporated with an accurate system model, a statistical noise model, and a prior model [12].

All images were anonymized and transferred to a post-processing 3-D console (SyngoVia VA30A; Siemens Healthcare).

### Image analysis

Images were analyzed independently by two radiologists (O.R. and M.H., with 25 years and 10 years of experience in pediatric lung CT, respectively), following the European Guidelines on Quality Criteria for CT. The ratings of the two readers were averaged. For all images, a dedicated lung convolution kernel (Bl57) was used, as recommended by the manufacturer. Images were interpreted in axial, coronal and sagittal orientation with 1-mm slice thickness using a multiplanar imaging tool (MM Reading, SyngoVia VA30A; Siemens Healthcare). Maximum- and minimum-intensity projections were allowed to be used at the discretion of the readers. The default window setting was center –600 HU and width 1,700 HU and could be individually adjusted by the readers.

We rated diagnostic confidence as well as detectability of the following anatomical structures on a 4-point Likert scale (1 unacceptable, 2 acceptable under limited conditions, 3 probably acceptable, 4 fully acceptable): medium-size and small pulmonary vessels, tertiary bronchi, lung fissures, lung parenchyma. We also rated suspicious lung lesions with respect to detectability, contrast and contour sharpness using the same 4-point Likert scale.

To assess image quality, we measured noise in the tracheal lumen on 1.0-mm-thick axial images of all datasets (FBP, ADMIRE 2/3/4). Ten randomly selected patients were evaluated ex ante to detect the optimal surface of the circular region of interest (ROI) with respect to the anatomical target regions. Thus, the defined size of ROI was 0.4 cm^2^ for older children and adolescents and 0.2 cm^2^ for smaller children. For each axial image, we performed and averaged three measurements. Image noise was defined as the standard deviation of the attenuation value.

### Radiation exposure and effective dose

Radiation exposure was assessed as volumetric CT dose index (CTDI_vol_) and dose–length product (DLP). Estimated effective dose (ED) was calculated as DLP·k, using an individual linear interpolation of the conversion factor reported in literature for chest CT at 100 kV between neonates (k_0_=0.0739 mSv/mGy·cm), 1-year-olds (k_1_=0.048 mSv/mGy·cm), 5-year-olds (k_5_=0.0322 mSv/mGy·cm), 10-year-olds (k_10_=0.0235 mSv/mGy·cm) and 18-year-olds (k_18_=0.0144 mSv/mGy·cm) as a function of days of age [8, 13].

### Statistical analysis

Statistical analysis was performed using SPSS software version 25 (IBM, Armonk, NY) and WINPEPI (Abramson JH, Hebrew University, Jerusalem). Values are given as mean ± standard deviation if normal distribution was assumed by Kolmogorov–Smirnov tests. Nominal variables were also expressed as frequencies. For multiple comparisons one-way analysis of variance (ANOVA) multiple comparison test with Bonferroni and Games–Howell post hoc pairwise comparisons were applied. All tests were performed two-sided, and *P*<0.05 was considered to be statistically significant. We calculated proportion of inter-rater disagreement and information-based measure of disagreement (IBMD). IBMD measures the level of disagreement between two or more observers. A value of 0 indicates no disagreement, whereas a value of 1 indicates total disagreement [14].

## Results

### Diagnostic confidence

Diagnostic confidence improved in all Sn groups with increasing strength levels of ADMIRE (Table 2). There was no significant difference in diagnostic confidence between the full-dose group reconstructed with ADMIRE 2 and the Sn96 group reconstructed with ADMIRE 4 (FD_ADM2_ vs_._ Sn96_ADM4_: *P*=0.092). Although differences between the FD_ADM2_ group and the Sn64_ADM4_ and Sn32_ADM4_ groups were significant (FD_ADM2_ vs_._ Sn64_ADM4_: *P*=0.008; FD_ADM2_ vs_._ Sn32_ADM4_: *P*<0.001), diagnostic confidence reached a Likert score >3 in the Sn64_ADM4_ group. This was not true for the Sn32_ADM4_ group (2.7). For further information see Table 2. The two readers disagreed in 50 of 224 ratings (22%, IBMD 0.10, 95% confidence interval [CI] 0.08–0.12).

**Table 2:** Diagnostic confidence and detectability of anatomical structures of different dose groups

Dose group		FD	Sn96	Sn64	Sn32	Relevant *P*-values^c^	
Diagnostic confidence	FBP	3.7±0.4	2.3±0.6	1.9±0.6	1.3±0.3^a,b^	FD_ADM2_ vs. Sn64_ADM4_:	*P*=0.008
	ADMIRE 2	3.8±0.5	2.7±0.4	2.5±0.4	1.8±0.4^a,b^	FD_ADM2_ vs. Sn32_ADM4_:	*P*<0.001
	ADMIRE 3		3.3±0.5	2.8±0.5	2.3±0.4^a,b^	Sn64_ADM4_ vs. Sn32_ADM4_:	*P*=0.058
	ADMIRE 4		3.3±0.5	3.2±0.4	2.7±0.6^a^		
Medium-size vessels	FBP	3.8±0.5	3.6±0.7	3.3±0.4	2.4±0.5^a,b^	FD_ADM2_ vs. Sn64_ADM4_:	*P*=1
	ADMIRE 2	3.8±0.4	3.7±0.6	3.6±0.4	3.0±0.6^a,b^	FD_ADM2_ vs. Sn32_ADM4_:	*P*=0.036
	ADMIRE 3		3.8±0.5	3.8±0.4	3.1±0.7^a,b^	Sn64_ADM4_ vs. Sn32_ADM4_:	*P*=0.007
	ADMIRE 4		3.8±0.4	3.9±0.2	3.4±0.7^b^		
Small vessels	FBP	3.5±0.7	2.3±0.5	2.0±0.5	1.3±0.4^a,b^	FD_ADM2_ vs. Sn64_ADM4_:	*P*=0.468
	ADMIRE 2	3.4±0.6	3.0±0.5	2.7±0.5	2.0±0.7^a,b^	FD_ADM2_ vs. Sn32_ADM4_:	*P*<0.001
	ADMIRE 3		3.1±0.6	3.1±0.4	2.1±0.4^a,b^	Sn64_ADM4_ vs. Sn32_ADM4_:	*P*=0.028
	ADMIRE 4		3.2±0.6	3.1±0.4	2.5±0.6^a,b^		
Tertiary bronchi	FBP	3.8±0.5	3.1±0.6	2.8±0.6	2.2±0.5^a,b^		
	ADMIRE 2	3.8±0.4	3.6±0.5	3.5±0.5	3.2±0.7	FD_ADM2_ vs. Sn64_ADM4_ vs. Sn32_ADM4_:	ANOVA *P*=0.132
	ADMIRE 3		3.8±0.4	3.8±0.4	3.3±0.7^a,b^		
	ADMIRE 4		3.8±0.4	3.7±0.4	3.5±0.5		
Lung fissures	FBP	3.3±0.8	2.2±0.6	2.2±0.5	1.3±0.5^a,b^	FD_ADM2_ vs. Sn64_ADM4_:	*P*=0.087
	ADMIRE 2	3.5±0.7	2.7±0.7	2.6±0.8	1.8±0.8^a,b^	FD_ADM2_ vs. Sn32_ADM4_:	*P*<0.001
	ADMIRE 3		2.8±0.8	2.8±0.6	2.0±0.6^a,b^	Sn64_ADM4_ vs. Sn32_ADM4_:	*P*=0.008
	ADMIRE 4		2.8±0.7	3.0±0.6	2.2±0.5^b^		
Lung parenchyma	FBP	3.5±0.6	2.0±0.7	1.6±0.5^b^	1.6±0.5^a^	FD_ADM2_ vs. Sn64_ADM4_:	*P*=0.001
	ADMIRE 2	3.6±0.5	2.7±0.5	2.6±0.5	1.8±0.4^a,b^	FD_ADM2_ vs. Sn32_ADM4_:	*P*<0.001
	ADMIRE 3		2.6±0.5	2.5±0.5	2.1±0.4^a^	Sn64_ADM4_ vs. Sn32_ADM4_:	*P*=1
	ADMIRE 4		2.9±0.5	2.9±0.4	2.7±0.5		

Ratings on a 4-point Likert scale (1 = unacceptable, 2 = acceptable under limited conditions, 3 = probably acceptable, 4 = fully acceptable) of the different dose groups. Values are given as mean ± standard deviation

*FBP* filtered back-projection, *FD* full dose, *Sn96/Sn64/Sn32* dose groups with tin prefiltration and different reference tube current time products (96/64/32 reference mAs, respectively)

^a^Values significantly lower compared to Sn96

^b^Values significantly lower compared to Sn64

^c^Relevant *P*-values in respect of the hypothesis

### Anatomical structures

**Fig. 1:**
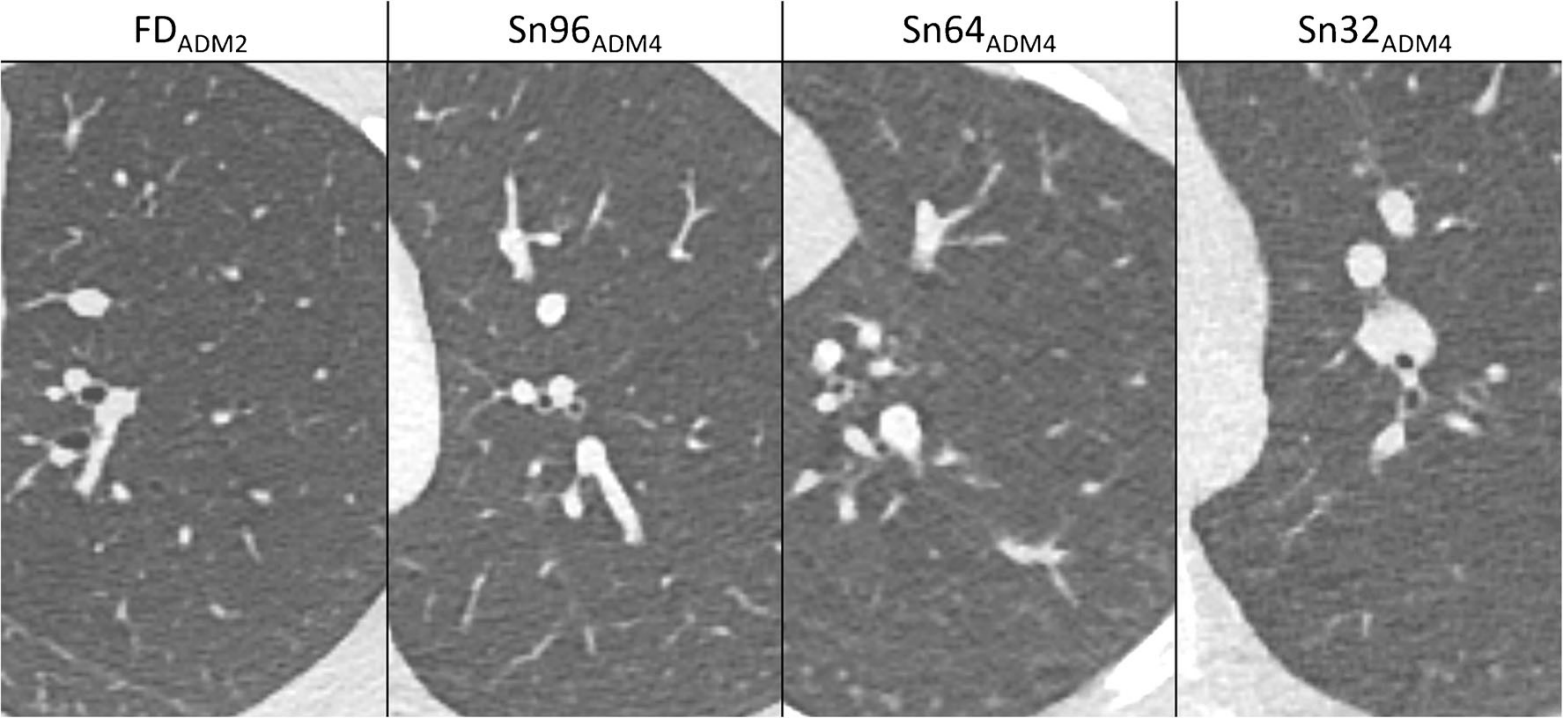
Examples of comparative detectability of anatomical structures in non-affected lung regions on axial CT slices. **FD**_**ADM2**_: full-dose, reconstruction with ADMIRE 2 in a 16-year-old girl with a prolonged course of pneumonia. **Sn96**_**ADM4**_: tin prefiltration, 96 reference mAs, ADMIRE 4 in a 12-year-old boy with atelectasis of the right upper lobe of unclear origin. **Sn64**_**ADM4**_: tin prefiltration, 64 reference mAs, ADMIRE 4 in a 15-year-old girl with suspected fungal pneumonia. **Sn32**_**ADM4**_: tin prefiltration, 32 reference mAs, ADMIRE 4 in a 14-year-old boy with prolonged course of pneumonia. The detectability of small anatomical structures is acceptable in the FD_ADM2_, Sn96_ADM4_ and Sn64_ADM4_ groups but is limited in the Sn32_ADM4_ group. *FD* full-dose group, *Sn96/Sn64/Sn32* groups with tin prefiltration at different reconstruction algorithms (filtered back-projection/ADMIRE 2/3/4)

In all Sn groups, detectability of anatomical structures improved with increasing strength levels of ADMIRE (Table 2). Compared to FD_ADM2_, values for Sn96_ADM4_, Sn64_ADM4_ and Sn32_ADM4_ were 3.4±0.6 vs. 3.2±0.6, 3.1±0.4 and 2.5±0.6 for small vessels; 3.8±0.4 vs. 3.8±0.4, 3.7±0.4 and 3.5±0.5 for tertiary bronchi; and 3.5±0.7 vs. 2.8±0.7, 3.0±0.6 and 2.2±0.5 for lung fissures. Except for lung parenchyma, no significant differences in detectability of anatomical structures were found between FD_AM2_ and Sn64_ADM4_. On the other hand, differences between Sn64_ADM4_ and Sn32_ADM4_ were statistically significant with the exception of tertiary bronchi and lung parenchyma. More detailed information regarding all evaluated anatomical structures and corresponding values of significance is described in Table 2. An example is given in Fig. 1. The two readers disagreed in 281 of 1,120 ratings (25%, IBMD 0.10, 95% CI 0.08–0.12).

### Suspicious lung lesions

A total of 231 lung lesions were identified on the CT examinations. Mean diameters of the lesions were 6.1±5.6 mm in the full-dose, 5.0±4.5 mm in the Sn96, 6.2±5.9 mm in the Sn64, and 7.6±6.6 mm in the Sn32 groups (ANOVA: *P*=0.095; Table 3). The lesions comprised subpleural, peribronchovascular or centrilobular nodules, mucoid impaction, tree-in-bud opacities, septal thickening, local ground-glass opacity, circumscribed consolidations, abscess formation, bronchiectasis, pneumatoceles and cavitations.

**Table 3:** Characteristics and evaluation of suspicious lung lesions of different dose groups

Dose group	FD_ADM2_	Sn96_ADM4_	Sn64_ADM4_	Sn32_ADM4_	*P*-value^a^
Number of lesions	53	61	64	53	
Lesions per patient	3.3	3.8	4.0	3.3	
Size (mm)	6.1±5.6	5.0±4.5	6.2±5.9	7.6±6.6	FD vs. Sn96 vs. Sn64 vs. Sn32: ANOVA *P*=0.095
Detectability	3.8±0.3	3.4±0.6	3.3±0.7	3.0±0.7	FD vs. Sn96/Sn64/Sn32: *P*<0.001 Sn96 vs. Sn64: *P*=0.985 Sn64 vs. Sn32: *P*=0.125 Sn96 vs. Sn32: *P*=0.020
Contrast	3.6±0.5	2.9±0.7	2.9±0.6	2.6±0.7	FD vs. Sn96/Sn64/Sn32: *P*<0.001 Sn96 vs. Sn64: *P*=1 Sn64 vs. Sn32: *P*=0.177 Sn96 vs. Sn32: *P*=0.345
Contour sharpness	3.5±0.6	2.9±0.8	2.8±0.7	2.3±0.7	FD vs. Sn96/Sn64/Sn32: *P*<0.001 Sn96 vs. Sn64: *P*=0.945 Sn96/Sn64 vs. Sn32: *P*<0.001

Detectability, contrast and contour sharpness of lesions are rated on a 4-point Likert scale (1 = unacceptable, 2 = acceptable under limited conditions, 3 = probably acceptable, 4 = fully acceptable)

*ANOVA* analysis of variance, *FD* full dose, *FD*_*ADM2*_ full dose, reconstruction with ADMIRE 2, *Sn96*_*ADM4*_*/Sn64*_*ADM4*_*/Sn32*_*ADM4*_ tin prefiltration with 96/64/32 reference mAs, respectively, reconstruction with ADMIRE 4

^a^ Post-hoc pairwise comparisons are displayed when ANOVA *P*<0.05

**Fig. 2:**
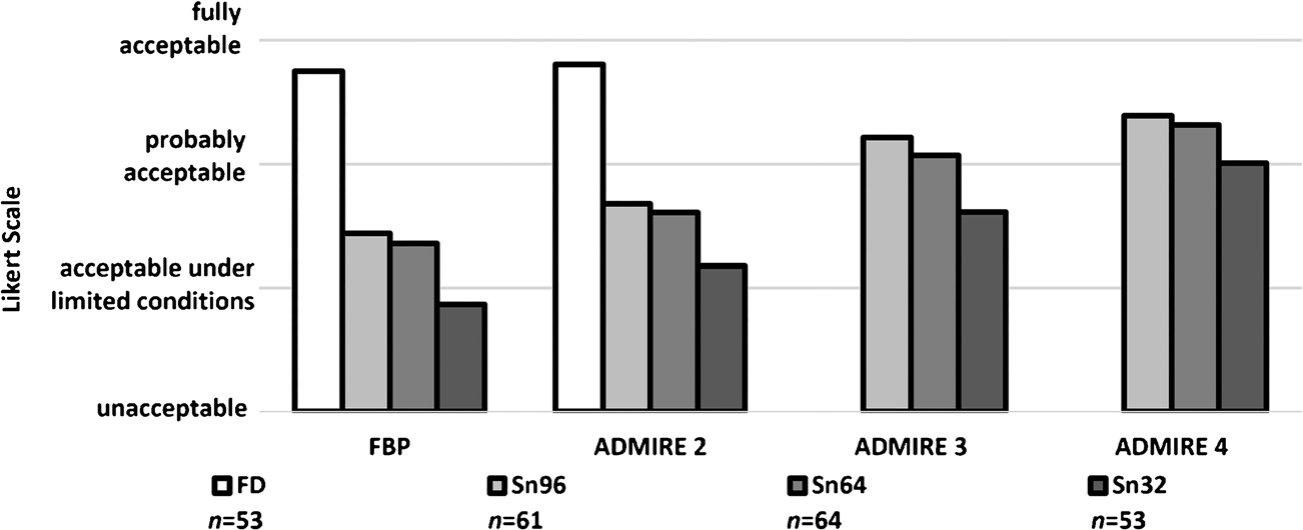
Detectability of suspicious lung lesions (*n*) in the different dose groups rated on a 4-point Likert scale. *FBP* filtered back-projection, *FD* full-dose group, *Sn96/Sn64/Sn32* groups with tin prefiltration at different reconstruction algorithms (FBP/ADMIRE 2/3/4)

**Fig. 3:**
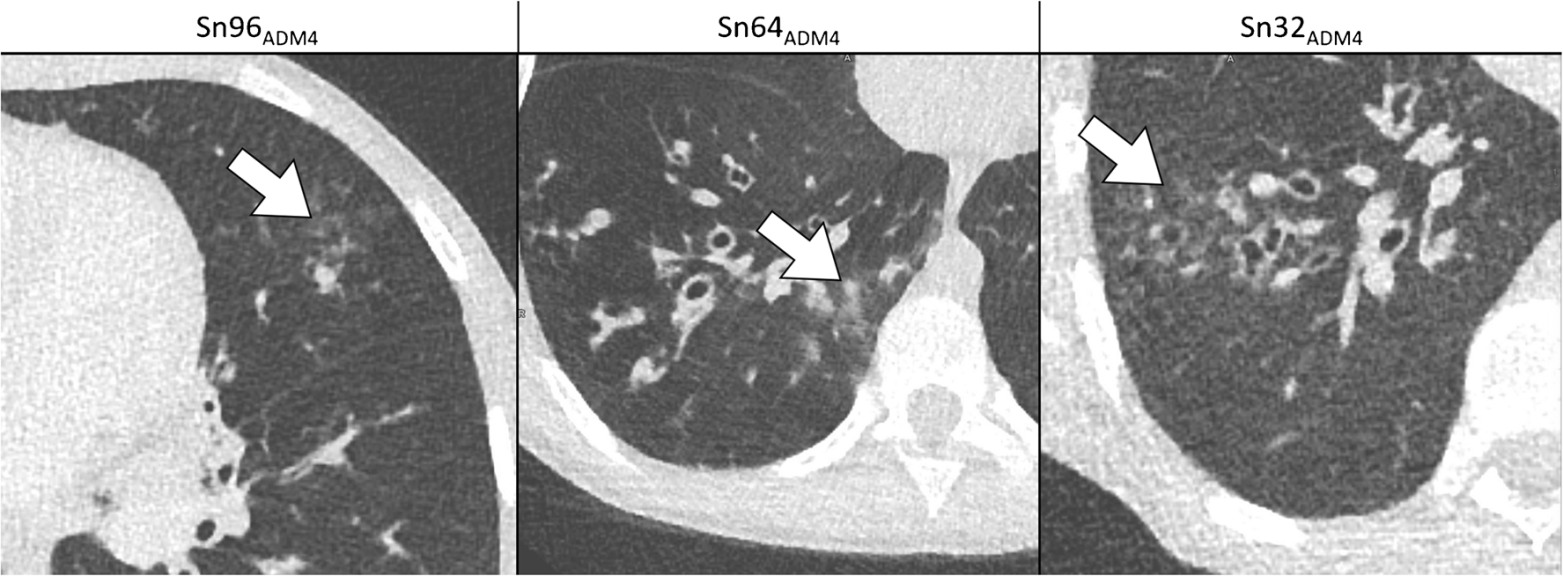
Examples of comparative detectability of circumscribed consolidations (*arrows*) of cystic fibrosis on axial CT slices. **Sn96**_**ADM4**_: tin prefiltration, 96 reference mAs, ADMIRE 4 in a 14-year-old girl. **Sn64**_**ADM4**_: tin prefiltration, 64 reference mAs, ADMIRE 4 in a 10-year-old boy. **Sn32**_**ADM4**_: tin prefiltration, 32 reference mAs, ADMIRE 4 in a 17-year-old boy. Detectability of circumscribed consolidations is acceptable in the Sn96_ADM4_ and Sn64_ADM4_ groups. In the Sn32_ADM4_ group, interfering noise causes a significant loss of contour sharpness, and detectability is significantly restricted. *Sn96/Sn64/Sn32* groups with tin prefiltration at ADMIRE 4 reconstruction algorithm

Concerning detectability, contrast and contour sharpness of lesions, differences between the FD_ADM2_ group and all Sn_ADM4_ groups turned out to be significant in terms of statistics (*P*<0.001; Table 3). Nevertheless, a Likert score value clearly >3 was reached in the Sn96_ADM4_ and Sn64_ADM4_ groups regarding lesion detectability (3.4 and 3.3; Fig. 2). Moreover, Sn64_ADM4_ only marginally missed a score value of 3 points concerning contrast (2.9) and contour sharpness (2.8). Compared to the Sn64_ADM4_ group, there were lower score values in the Sn32_ADM4_ group, being significant concerning contour sharpness (2.3, *P*<0.001; Table 3). An example is given in Fig. 3. The two readers disagreed in 648 of 2,454 ratings (26%, IBMD 0.12, 95% CI 0.10–0.13).

### Image quality

**Fig. 4:**
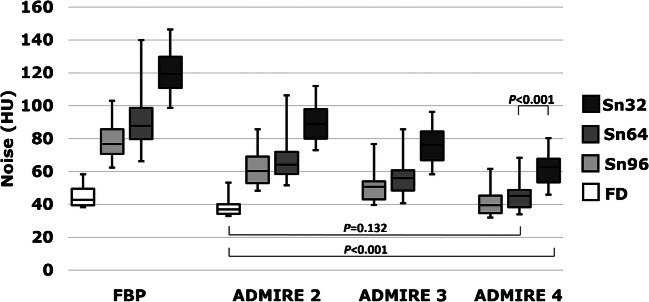
Boxplot represents noise measured in the tracheal lumen of patients of the different dose groups. Boxes represent the 25% and 75% quartiles, whiskers the minimum and maximum values. Additionally, significance levels of post hoc pairwise comparisons are displayed for FD_ADM2_ vs. Sn64_ADM4_/Sn32_ADM4_ and Sn64_ADM4_ vs. Sn32_ADM4_. Noise did not statistically differ between FD_ADM2_ group and Sn64_ADM4_ group (*P*=0.132), whereas noise was significantly higher in the Sn32_ADM4_ group compared to the FD_ADM2_/Sn64_ADM4_ groups (*P*<0.001). *FD* full-dose group, *Sn96/Sn64/Sn32* groups with tin prefiltration at different reconstruction algorithms (filtered back-projection/ADMIRE 2/3/4)

**Fig. 5:**
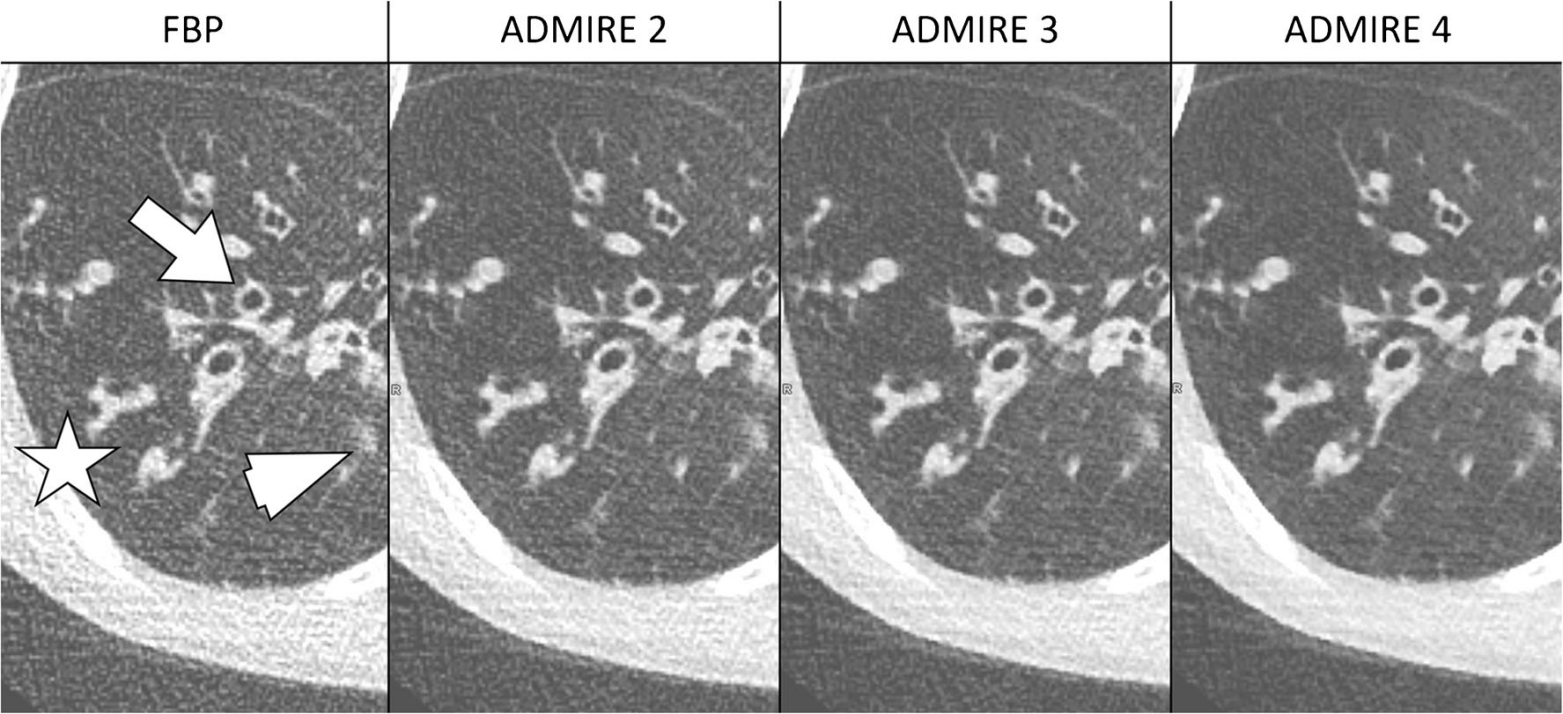
Influence of reconstruction algorithms (filtered back-projection [FBP], ADMIRE 2/3/4) on image quality and noise in a 10-year-old boy with cystic fibrosis from the Sn64 group (tin prefiltration, 64 reference mAs). Axial CT images depict bronchiectasis (*arrow*), mucoid impaction (*asterisk*) as well as circumscribed consolidations (*arrowhead*). Compared to FBP, noise decreases with increasing strength of ADMIRE

Regarding tin prefiltration, lowering of the reference mAs effected an increase of noise. With increasing strength levels of ADMIRE, noise significantly decreased in all study groups (Fig. 4). Corresponding values for FBP and ADMIRE 2, 3 and 4 were 78.3±12.1 HU, 61.3±10.5 HU, 50.3±9.4 HU and 40.6±8.0 HU in the Sn96 group; 92.3±18.5 HU, 67.3±12.9 HU, 57.1±11.6 HU and 45.7±9.3 HU in the Sn64 group; and 120.9±15.4 HU, 90.2±12.7 HU, 75.7±11.0 HU and 61.5±9.3 HU in the Sn32 group (*P*<0.001). An example is given in Fig. 5. Noise value of the Sn64_ADM4_ group did not statistically differ from that in the FD_ADM2_ group (45.7 vs. 38.8 HU, *P*=0.132; Fig. 4). On the other hand, noise was significantly higher in the Sn32_ADM4_ group compared to the FD_ADM2_ group (61.5 vs. 38.8 HU; *P*<0.001) and even to the Sn64_ADM4_ group (61.5 vs. 45.7 HU; *P*<0.001).

### Radiation exposure and effective dose

Compared to the full-dose group, the use of tin prefiltration led to a significantly lower radiation exposure in the Sn96, Sn64 and Sn32 groups. Mean CTDI_vol_ was 2.17±1.2 mGy vs. 0.31±0.14 in the Sn96 group, 0.24±0.10 mGy in Sn64, and 0.13±0.09 mGy in Sn32 (full dose vs. Sn_all_: *P*<0.001). Corresponding mean DLP was 70.3±44.0 mGy·cm in the FD group vs. 10.5±5.7 mGy·cm in the Sn96 group, 8.2±3.4 mGy·cm in Sn64, and 3.9±2.7 mGy·cm in Sn32 (*P*<0.001). Consequently, this led to a mean effective dose (ED) of 1.26±0.54 mSv in the FD group vs. 0.21±0.07 mSv in the Sn96 group, 0.14±0.05 mSv in Sn64, and 0.07±0.04 mSv in Sn32 (*P*<0.001). Among others, dose reduction was statistically significant between full-dose and Sn64 groups, as well as between Sn64 and Sn32 groups (*P*<0.001). Effective dose was reduced to 16.7%, 11.1% and 5.5% in the Sn96, Sn64 and Sn32 groups, respectively, compared to the full-dose group (Table 4).

**Table 4:** Radiation dose exposure and estimated effective dose among different dose groups

Dose group	FD	Sn96	Sn64	Sn32	*P*-value^a^
CTDI_Vol_ (mGy)	2.17±1.23	0.31±0.14	0.24±0.10	0.13±0.09	FD vs. Sn96/Sn64/Sn32: *P*<0.001 Sn64 vs. Sn32: *P*=0.008
DLP (mGy·cm)	70.3±44.0	10.5±5.7	8.2±3.4	3.9±2.7	FD vs. Sn96/Sn64/Sn32: *P*<0.001 Sn64 vs. Sn32: *P*=0.002
ED (mSv)	1.26±0.54	0.21±0.07	0.14±0.05	0.07±0.04	FD vs. Sn96/Sn64/Sn32: *P*<0.001 Sn64 vs. Sn32: *P<*0.001
Reduction of ED (percentage value of FD)		16.7%	11.1%	5.5%	

Values of volumetric CT dose index (CTDI_Vol_), dose–length product (DLP) and effective dose (ED) are given as mean ± standard deviation

*FD* full-dose group, *Sn96/Sn64/Sn32* groups with tin prefiltration and different reference tube current time products (96/64/32 reference mAs, respectively)

^a^ Significant differences (*P*<0.05) were found between FD group and all Sn groups, but also between Sn64 and Sn32 groups

## Discussion

In our retrospective study, pediatric lung dual-source CT examinations with spectral shaping led to significantly lower radiation exposure compared to a full-dose protocol. In terms of statistics, dose lowering to about 10% by using the Sn64 protocol caused reduction in diagnostic confidence. Nevertheless, acceptable Likert score values >3 were achieved for diagnostic confidence as well as detectability of lung lesions when ADMIRE 4 was performed. Simultaneously, there was no significant deterioration of detectability of most anatomical structures, and noise value did not statistically differ from the full-dose group.

There was a significant reduction of radiation exposure between the Sn64 and Sn32 groups. However, further dose reduction to about 5% of the full-dose group by using the Sn32 protocol caused significant loss of contour sharpness of lung lesions compared to the Sn64 group. Even when ADMIRE 4 was performed, visualization of the majority of anatomical structures was significantly reduced. Diagnostic confidence worsened, and noise significantly increased.

In the last few years, several studies proved the potential of lung CT to deliver adequate image quality when protocols with reduced dose were used [14–16]. In a study by Kroft et al. [15], mean perceived confidence for diagnosis was 98% for lung CT examinations with a mean effective dose of 0.07 mSv. Ebner et al. [16] investigated chest phantoms with artificial lung nodules between 5 mm and 12 mm at a mean dose level of 0.13 mSv. Sensitivity for nodule detection was 96.2% [16]. According to Neroladaki et al. [17], model-based iterative reconstruction allows secure detection of pulmonary nodules in adults at a radiation dose level of 0.16 mSv.

To our knowledge, studies investigating the effect of tin prefiltration on dose reduction are still rare in the pediatric population. Weis et al. [18] compared a 100-kV pediatric chest CT protocol using spectral shaping (Sn100 kV) with a 70-kV standard protocol. Significant dose reduction up to 0.21 mSv and superior subjective image quality of lung structures was achieved with the Sn100-kV protocol. Consequently, their dose results resemble the mean radiation dose of the Sn96 group in our study. In a phantom study, Martini et al. [19] analyzed solid and subsolid lung lesions with low-dose protocols using tin prefiltration. Resulting effective doses were comparable to ours (0.14 mSv at 1/8th and 0.05 mSv at 1/20th of standard dose). They reached diagnostic image quality when using ADMIRE Levels 3 or 5. Bodelle et al. [5] evaluated the effect of spectral shaping on image quality and effects on radiation parameters using a single-source 100-kV pediatric chest protocol. With the use of tin prefiltration, increase of effective tube current up to a factor of 10 provided similar image quality with comparable noise at equivalent dose compared to the standard protocol without spectral filtration. Without spectral shaping, CTDI was 3 times higher compared to our Sn96 group, whereas it was still 2.5 times higher when tin prefiltration was added.

This study has some limitations. Because of its retrospective design, patients’ age varied from 1.3 years to 18.0 years, with only few small children being included. Therefore our assertions might not be representative for the last-mentioned. Further research is needed in this area, for example with regard to pulmonary metastases in small children with cancer, which was not part of our study. Moreover, we cannot provide sensitivity of lung lesion detection because no internal reference standard was available for comparison. Instead, we evaluated diagnostic confidence and detectability of both anatomical lung structures and suspicious lung lesions. Sensitivity regarding detection of small pulmonary lesions with reduced-dose protocols is known to be high. Messerli et al. [20] detected lung nodules in adults with a sensitivity of 91.2% using a low-radiation-dose protocol comparable to our Sn64 protocol. In a phantom study performed by Grodic et al. [21], sensitivity of pulmonary nodule detection was 94% in a reduced-dose group with tin prefiltration (1/10th of standard dose) and ADMIRE 5. Although results of sensitivity given from these studies cannot be assigned to our collective, they at least tend to support the validity of our findings.

## Conclusion

In pediatric lung dual-source CT with spectral shaping, dose reduction to about 10% of a full-dose protocol still enables acceptable diagnostic quality when image reconstruction is performed with ADMIRE 4.
